# Corruption risks in health procurement during the COVID-19 pandemic and anti-corruption, transparency and accountability (ACTA) mechanisms to reduce these risks: a rapid review

**DOI:** 10.1186/s12992-023-00994-x

**Published:** 2023-11-24

**Authors:** Kari A. Griffore, Andrea Bowra, Sara J.T. Guilcher, Jillian Kohler

**Affiliations:** 1 World Health Organization Collaborating Centre for Governance, Accountability, and Transparency in the Pharmaceutical Sector, 144 College Street, Toronto, ON M5S 3M2 Canada; 2 Leslie Dan Faculty of Pharmacy, Canada University of Toronto, 144 College Street, Toronto, ON M5S 3M2 Canada; 3 Dalla Lana School of Public Health, University of Toronto, 155 College St, Toronto, M5T 3M7 Canada

**Keywords:** Corruption, Global health, Substandard medicines, Falsified medicines, COVID- 19

## Abstract

**Background:**

Health systems are often susceptible to corruption risks. Corruption within health systems has been found to negatively affect the efficacy, safety, and, significantly, equitable distribution of health products. Enforcing effective anti-corruption mechanisms is important to reduce the risks of corruption but requires first an understanding of the ways in which corruption manifests. When there are public health crises, such as the COVID-19 pandemic, corruption risks can increase due to the need for accelerated rates of resource deployment that may result in the bypassing of standard operating procedures.

**Main body:**

A rapid review was conducted to examine factors that increased corruption risks during the COVID-19 pandemic as well as potential anti-corruption, transparency and accountability (ACTA) mechanisms to reduce these risks. A search was conducted including terms related to corruption, COVID-19, and health systems from January 2020 until January 2022. In addition, relevant grey literature websites were hand searched for items. A single reviewer screened the search results removing those that did not meet the inclusion criteria. This reviewer then extracted data relevant to the research objectives from the included articles. 20 academic articles and 17 grey literature pieces were included in this review. Majority of the included articles described cases of substandard and falsified products. Several papers attributed shortages of these products as a major factor for the emergence of falsified versions. Majority of described corruption instances occurred in low- and middle-income countries. The main affected products identified were chloroquine tablets, personal protective equipment, COVID-19 vaccine, and diagnostic tests. Half of the articles were able to offer potential anti-corruption strategies.

**Conclusion:**

Shortages of health products during the COVID-19 pandemic seemed to be associated with increased corruption risks. We found that low- and middle-income countries are particularly vulnerable to corruption during global emergencies. Lastly, there is a need for additional research on effective anti-corruption mechanisms.

## Introduction

On March 11th, 2020, the World Health Organization (WHO) declared the outbreak of the novel COVID-19 virus to be a pandemic [1]. As a result of the pandemic, there was a sudden increase globally in demand for health products, supply shortages, and fast-tracked approvals for COVID-19 related treatments [2]. Indeed, a major challenge for governments and international organizations during the pandemic has been ensuring the sufficient production and distribution of medical products and pharmaceuticals globally [3]. An example of efforts made by international organizations to ensure sufficient distribution of supplies includes the WHO in March of 2020 working with governments and industry to boost production of, and secure allocations to, critically affected and at-risk countries following severe personal protective equipment (PPE) shortages [4].

Health systems are often at risk for corruption, especially during global health emergencies, such as the COVID-19 pandemic [5]. Corruption is defined by Transparency International as “the abuse of entrusted power for private gain” [6]. Examples of corruption include but are not limited to production and distribution of substandard and falsified medicines, embezzlement of funds and resources, and a lack of transparency in governance [7]. Corruption within health systems has drastic effects on the equitable distribution and quality of essential medical supplies and pharmaceuticals, that is particularly concerning during a global health emergency, as equity-seeking groups are most at risk of experiencing the worst effects of corruption [8].

Corruption and public emergencies essentially feed off each other to exacerbate corruptions risks [5]. In a state of emergency, governments must make decisions to fulfil public health needs while facing the pressure to secure products as quickly as possible, sometimes with financial interests complicating decisions [5, 8]. Many states have laws in place which allow for removal of certain governance and transparency rules surrounding procurement in order for accelerated action to occur during a state-of-emergency [9]. However, what is concerning is that this relaxing of rules is often not accompanied by necessary monitoring mechanisms which further heightens the risk of emergence of corruption [9].

Therefore, COVID-19 has highlighted the need for effective anti-corruption, transparency, and accountability (ACTA) measures to be put in place globally. ACTA mechanisms are necessary to improve health care efficacy by ensuring that funds and resources allocated to improve the health of populations fulfil their intended purposes [10, 11]. Increasing efforts have been made over the recent years by international organizations such as the Global Fund, the UNDP, the WHO, and the World Bank to implement ACTA mechanisms within their governance structure [12]. Transparency is a key factor in minimizing corruption risks as it lowers the information barrier, allowing for increased public scrutiny and monitoring [13]. In addition, accountability is an important component of anti-corruption strategies as it demands that those in power explain and take responsibility for their actions [14]. Implementation of effective anti-corruption measures is only possible if we first gain an understanding of the way in which corruption manifests and the factors that enable it [6].

To advance understanding of corruption during COVID-19 pandemic, this rapid review employs Vian’s Framework of Corruption in the Health Sector (2008/2020) to achieve the following objectives: (1) identify factors that may foster corruption at the international and national levels and impact access to COVID-19 vaccines, PPE, and medicines; and, (2) identify ACTA mechanisms that can reduce the risk of corruption in procurement and distribution processes thereby ensuring better access to COVID-19 medicines, PPE, and vaccines; and 3) examine the barriers and facilitators to implementing ACTA mechanisms in the health sector during the COVID-19 pandemic [14, 15].

## Methods

**Fig. 1:**
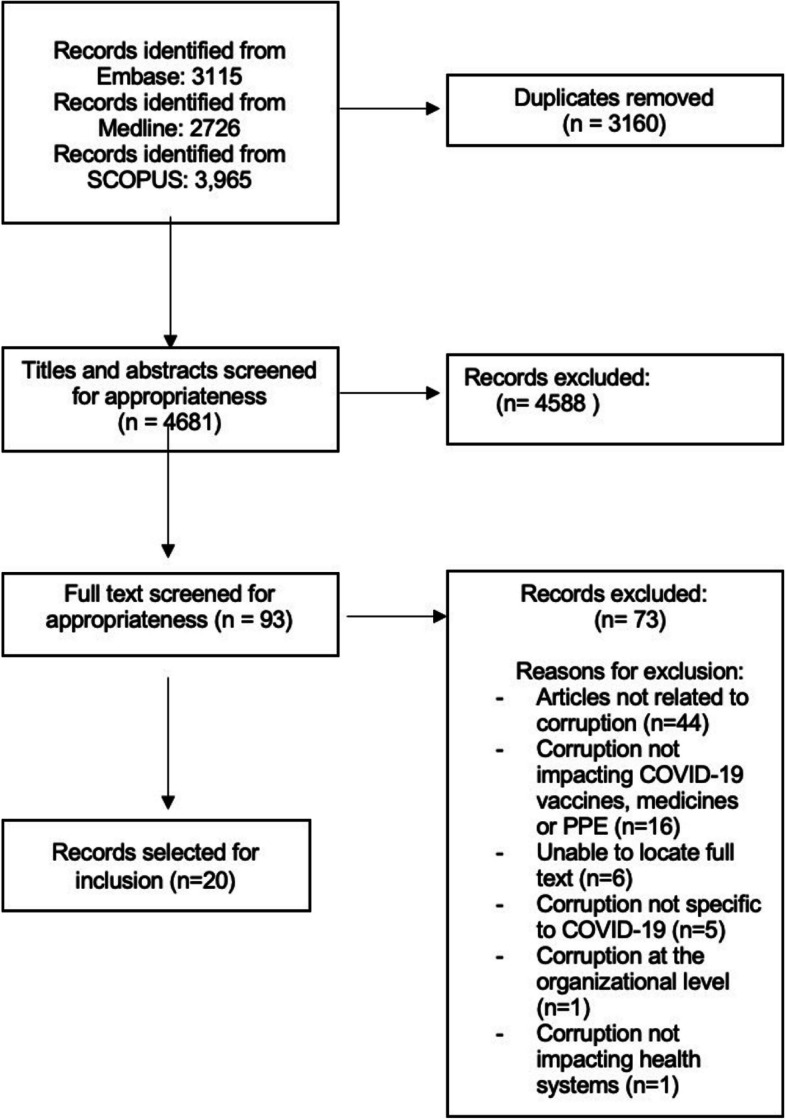
PRISMA chart

This research was guided by Vian’s 2008/2020 Framework of Corruption in the Health Sector (see Fig. 1) that explains factors that influence corruption in the health sector specifically [14, 15]. To address objective 1, this paper draws heavily on the left column of the framework – factors that increase opportunities for corruption. These factors include health care system and structure; monopoly; discretion; accountability; citizen voice; transparency; and enforcement [15]. We also draw on Vian’s 2020 update which identifies typologies and frameworks for addressing corruption in the health sector to achieve objectives 2 and 3 in identifying ACTA mechanisms applied or proposed to reduce corruption risks and their barriers or facilitators to implementation. Vian’s (2020) typologies include corruption frameworks, transparency frameworks, accountability frameworks, anti-corruption approaches, risk assessments, transparency interventions, community monitoring, whistleblowing, audits, systems-level approaches, standardized monitoring, and addressing under-resourcing [14].

The methodology used for this project is a rapid literature review, which provides actionable and relevant evidence within an accelerated timeframe [16]. In a rapid review, modifications are made to a knowledge synthesis review protocol to streamline the process by measures such as using a targeted research question and use of one reviewer for study selection and/or data extraction [17, 18]. This is an appropriate methodology for COVID-19 related questions, as timely reviews are of the utmost importance to inform health policy and systems recommendations such as in the case of enabling a call to action for policymakers during the COVID-19 pandemic [16].

Ovid Medline, Ovid Embase, and SCOPUS were searched for literature on corruption surrounding COVID-19 vaccines, PPE, and medicines with the guidance of a librarian. Key words searched included COVID-19, health systems, and corruption. The full list of search terms can be found in Table 1. Our inclusion criteria included articles published in the English language that contained information on corruption within the health system specifically related to COVID- 19 vaccines, medicines, or PPE, at the state/provincial, national, or international level. All publication types and study designs in which the full text was retrievable were included. Articles published after January 2020 up until the date of our search (January 17th, 2022) were included.

**Table 1:** Literature search strategy

Search	Query
#1	Corrupt*.ti,ab. OR anticorrupt*.ti,ab. OR anti-corrupt*.ti,ab. OR fraud*.ti,ab. OR falsifi*.ti,ab. OR embezzl*ti,ab. OR bribe*.ti,ab. OR integrity.ti,ab. OR transparen*.ti,ab. OR accountability.ti,ab. OR substandard.ti,ab. OR conflict*.ti,ab. OR illicit enrichment.ti,ab. OR conceal*.ti,ab. OR exploit*.ti,ab. OR abus*.ti,ab. OR informal pay*.ti,ab. OR kickback*.ti,ab. OR collusion*.ti,ab. OR pay off*.ti,ab. OR conflict interest.ti,ab. OR *Fraud/ OR *substandard drug/ OR *”Conflict of Interest”/
#2	Covid*.ti,ab. OR corona*.ti,ab. OR SARS-COV-2.ti,ab. OR *COVID-19/ OR *COVID-19 Testing/ OR *COVID-19 Vaccines/ OR SARS-COV-2/
#3	Drug industry.ti,ab. OR pharmaceutical industry.ti,ab. OR health*.ti,ab. OR health system*.ti,ab. OR pharm*.ti,ab. OR medicine*.ti,ab. OR health policy.ti,ab. OR *Drug Industry/ OR *Health Care Sector/ OR *Health Policy/
#4	#1 AND #2 AND #3

Limited to January 2020-present, English language

The search was run on January 17, 2022. Articles were imported into the platform Covidence and duplications were removed. One reviewer (KG) evaluated titles and abstracts and excluded any which were obviously irrelevant. Full texts of remaining citations were obtained, KG reviewed these, excluding any that did not meet inclusion criteria. Data extraction was carried out by KG.

A targeted grey literature search was done between May 16 – June 9th, 2022 on the following websites: WHO, the World Bank Group, the U4 Anti-Corruption Resource Centre, Transparency International, the UNODC, the UNDP, UNICEF, Interpol, The Justice Project, and the OECD. Searches were performed using the same search strategy used for the academic literature and identical inclusion criteria were applied. Data extraction from grey literature sources was performed by KG. The grey literature search was done to identify additional instances of corruption that occurred during the pandemic and related preventative mechanisms as the COVID-19 pandemic was a recent event at the time of our search and it is probable that many of these instances would not be reflected in the academic literature at this point in time.

## Results

The database search yielded a total of 4681 citations after duplicates were removed. The titles and abstracts of these articles were screened and 4588 were deemed irrelevant for our review and therefore excluded. The remaining 93 papers were selected for further assessment by full text screening. The PRISMA diagram can be found in Fig. 1, including reasons for exclusion within the full text screening stage. Fourteen out of 20 papers referred to corruption in low- and middle-income countries and 4 out of 20 discussed corruption issues in high-income countries. The remainder of articles did not highlight country-specific examples of corruption and instead included mentions of more general global corruption issues or mechanisms of combatting corruption globally. In addition, 17 pieces of grey literature were identified. Of the instances of corruption revealed in the grey literature, 4 cases occurred in low-income countries, 7 in middle-income countries, and 7 in high-income countries. Most cases of corruption identified were incidents of substandard and/or falsified products (*n* = 25). The main products that were implicated were hydroxychloroquine/chloroquine (*n* = 8), PPE (*n* = 10), COVID-19 vaccines (*n* = 7), and diagnostic tests (*n* = 2). The remaining types of corruption identified were collusion (*n* = 4), embezzlement (*n* = 2), fraud (*n *= 4), and concealment (*n* = 1).

Within the grey literature, the WHO published multiple medical product alerts over the course of the COVID-19 pandemic warning of falsified medical products globally. The WHO published a medical product alert in April 2020 after receiving 14 reports of confirmed falsified chloroquine products in Burkina Faso, Cameroon, Democratic Republic of Congo, France, and Niger [19]. Subsequent medical product alerts were published in August 2021 and May 2022 alerting the public of cases of confirmed falsified remdesivir in Mexico, Guatemala, and India [20, 21]. In addition, in March 2021, the WHO published an alert regarding confirmed falsified COVID-19 vaccines in Mexico that were being administered to patients outside of authorized vaccination programs [22]. Subsequent product alerts were published by WHO regarding confirmed falsified COVISHIELD and Pfizer-BioNTech COVID-19 vaccines in March, August, and November 2021 in Uganda, Myanmar, India, and Iran [22–24].

### Factors increasing opportunities for corruption

**Fig. 2:**
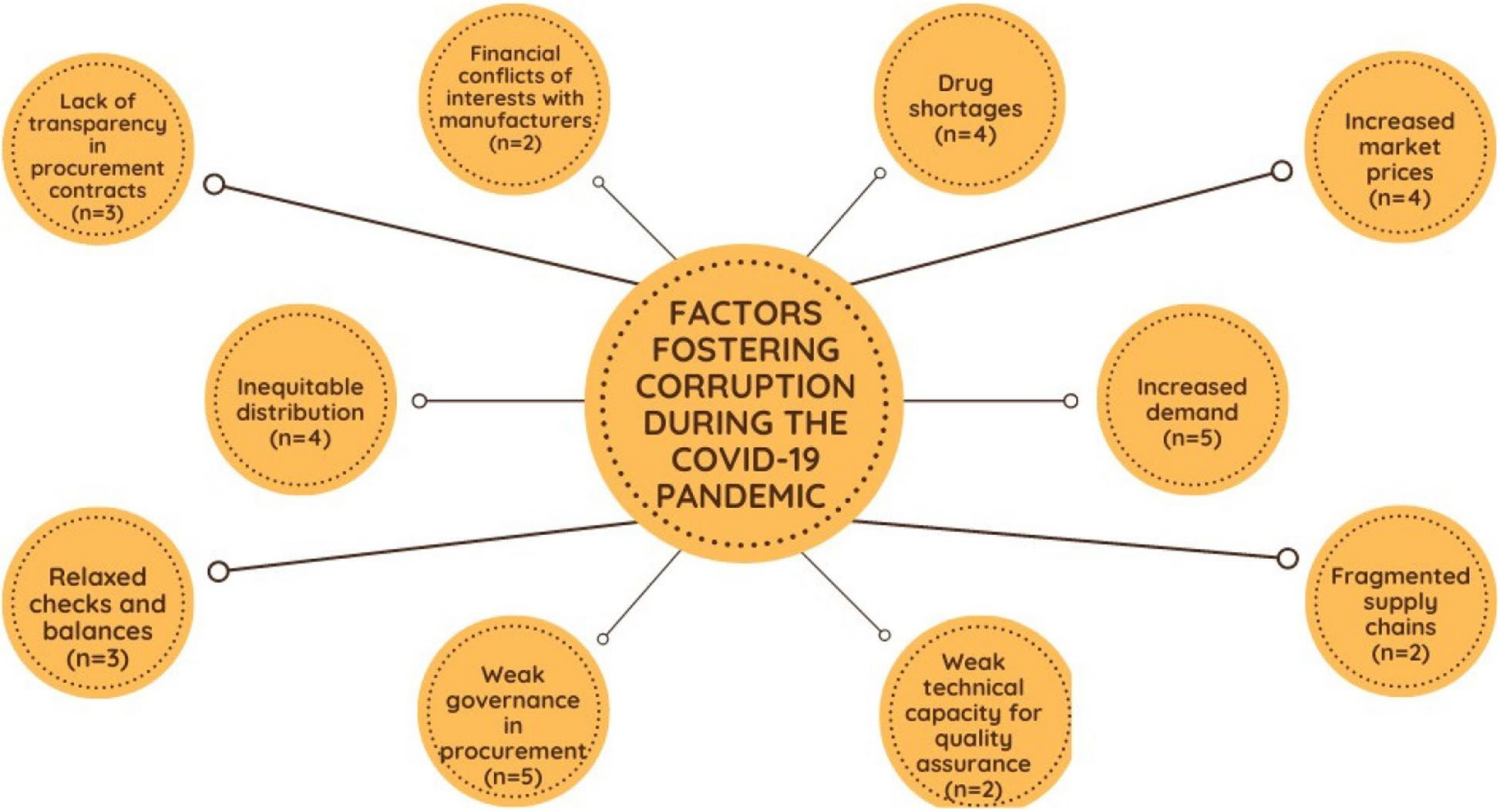
Factors facilitating corruption during the COVID-19 pandemic

Of the 20 peer-reviewed articles analyzed in this review, all highlighted factors that facilitated corruption. An overview can be seen in Fig. 2. Though there was significant overlap across identified factors, of Vian’s seven factors that increase opportunities for corruption, those most identified in the literature were (1) health care system and structure, (2) discretion, and (3) transparency and accountability. One additional factor was added to Vian’s (2008) framework, demand, since this factor was identified repeatedly in documents as fostering the production and distribution of substandard and falsified medical products [15].

### Health care system and structure

The most prominent factor identified as facilitating opportunities for corruption during the COVID-19 pandemic was a country’s health care system and structure. For example, Gnegal (2020) and Tesfaye (2021) indicated that the emergence of falsified medicines was especially prominent in low- and middle-income countries because of limited access to medicines, limited technical capacity and financial resources for quality assurance, and weak pharmaceutical governance [25, 26]. Further, Kohler and Wright (2020) and Moeshoeshoe (2022) noted that falsified vaccines may have been able to surface due to lack of anti-counterfeiting strategies and weak governance [27, 28].

Echoing the academic literature, online publications by the UNODC, the OECD, World Justice Project, and Transparency International highlighted several factors that led to increased corruption risks during the COVID-19 pandemic including relaxed procurement procedures and a lack of oversight of these processes. A UNODC policy brief noted that the increased emergence of falsified and substandard goods is parallel to relaxed procurement procedures in response to increased demand for products such as PPEs and COVID-19 vaccines [29]. In addition, a 2020 OECD brief highlighted that emergency procurement processes elevate corruption risks due to incomplete documentation, making it difficult for bodies to conduct audits. The brief also highlighted that risks are heightened in developing countries where market gaps and inconsistencies in public procurement were already prevalent prior to the pandemic [30]. Lastly, a brief published by U4/Transparency International in April 2021 highlighted several risk factors for corruption during the COVID-19 pandemic including a lack of clear legal framework needed to counter corruption such as effective conflict of interest laws, free access to public information laws, and whistleblower protection [31].

### Discretion

Discretion, which Vian (2008/2020) employs to describe the decision-making power of a government official or other person in a position of power, was identified as well as a factor increasing opportunities for corruption during the COVID-19 pandemic [14, 15]. Abbasi (2020) attributed the procurement of substandard tests partly to the presence of a financial conflict of interest between government officials, who had sole decision-making authority, and diagnostic test manufacturers [32]. Mottay (2020) and Jarrett (2020) similarly identified emergence of substandard face masks [33, 34]. Both identified that substandard PPE products may surface due to government officials deciding to engage with private donors and purchase products from unofficial retailers [33, 34].

### Transparency and accountability

Within the field of anti-corruption, transparency and accountability are closely related because transparency relates to the active disclosure of decision-making information so that officials can be held to account by the public they serve. The lack of both transparency and accountability were identified in the literature as increasing opportunities for corruption in health systems during COVID-19. Srivastava (2021) attributed the emergence of falsified vaccines to vaccine inequity and lack of transparency [35]. Additionally, policy briefs published by the World Justice Project and UNDP in September 2020 noted relaxation of procurement oversight and enforcement and a lack of safeguards in transparency and accountability mechanisms led to increased corruption risks in the procurement of COVID-19 supplies [36, 37].

### Demand

Several papers identified substandard and falsified chloroquine tablets in African countries including Cameroon, the Democratic Republic of Congo (DRC), Kenya, and Niger [25, 26, 38, 39]. Belayney (2020), Gnegal (2020), Tesfaye (2021), and Waffo Tchounga (2021) attributed the emergence of this falsified chloroquine to the increased demand for these medications due to the off-label use of chloroquine for the treatment of COVID-19 leading to shortages and increased market prices [25, 26, 39, 27, 34]. Jarret (2020) highlighted that the production of these falsified products can be highly profitable due to the high demand for and limited supply of vaccines during the COVID- 19 pandemic [34].

### Anti-corruption, transparency, and accountability (ACTA) mechanisms

Of the 20 peer-reviewed studies, 10 discussed potential anti-corruption, transparency, and accountability (ACTA) mechanisms that could be implemented to reduce corruption risks in the procurement and distribution of health products. Of Vian’s typologies for addressing corruption in the health sector (2020), the literature identified anti-corruption approaches, standardized monitoring, transparency interventions, accountability interventions, and systems-level approaches [14]. It is important to note that these typologies are not discreet and there is therefore overlap across categories. The identified mechanisms included the establishment of screening technologies such as Raman spectroscopy or chromatography that can detect substandard medicines (standardized monitoring), transparency in procurement processes (transparency interventions), punitive sanctions for those that sell or distribute falsified products (accountability interventions), and creating legislation and systems in place prior to health emergencies (systems-level approaches).

### Anti-corruption approaches

Within the grey literature, 7 documents from international organizations provided guidance on measures to reduce corruption during the COVID-19 pandemic. A guidance note published by the World Bank Group in April 2020 offered possible anti-corruption mechanisms that may be beneficial during the COVID-19 pandemic [39]. This included that legislation and executive orders should be put in place to outline responsibilities for the oversight of the procurement of goods as well as activation of explicit processes for documenting procurement transactions [40]. Similarly, a May 2020 policy brief by OECD highlighted the need to create detailed guidelines on procurement strategies during crises including policies for audit and oversight [30]. The World Justice Project published a policy brief which reinforced the notion that regardless of how expedited procurement processes are, procurement should still be auditable and outline considerations justifying the expedited process [36].

In September 2020, the UNDP published a guidance note on transparency, accountability, and anti-corruption services that can be utilized towards COVID-19 response and recovery [37]. Priorities that were highlighted include strengthening of internal and external oversight and audit capacity within institutions [37]. In addition, this note highlighted the importance of encouraging participation of civil society to enhance procurement oversight [37]. A UNDP policy paper highlighted the need for the creation of a specialized committee with a strong anti-corruption mandate to oversee the prioritization, distribution and monitoring of vaccine programmes and act as a critical oversight body [37].

### Standardized monitoring

With respect to implementation of screening technologies, Gnegal and colleagues (2020) suggest utilization of simple and inexpensive screening technologies such as thin layer chromatography or Raman spectroscopy in low-and middle-income countries [25]. For example, the Ecumenical Pharmaceutical Network (EPN) is a non-profit organization that offers quality pharmaceutical services across Africa has monitored medicine quality in countries using thin layer chromatography. Use of this screening technology allowed for identification of falsified chloroquine tables in private and informal markets in Cameroon and the Democratic Republic of Congo [25].

### Transparency interventions

Several guidance publications highlighted the benefit that e-procurement platforms could have on reducing corruption risks in procurement processes. To begin, a policy brief published by the OECD in May 2020 suggested leveraging e-procurement platforms to record transactions and create easily accessible tools to allow for public oversight of emergency procedures [30]. On April 7, 2021, a research brief from U4 and Transparency International underscored measures to mitigate corruption risks in the COVID-19 vaccine rollout [37]. This research brief encouraged the use of open contracting and e-procurement for COVID-19 to mitigate corruption risks [37]. Similarly, a UNDP policy paper highlighted the benefit that e-procurement and open contracting can have on reducing corruption risks [37].

### Systems-level approaches

Within the grey literature, 7 documents from international organizations provided guidance on measures to reduce corruption during the COVID-19 pandemic. A guidance note published by the World Bank in April 2020 offered possible mechanisms that may be beneficial during the COVID-19 pandemic [40]. This included that legislation and executive orders should be put in place to outline responsibilities for the oversight of the procurement of goods as well as activation of explicit processes for documenting procurement transactions [40]. Similarly, a May 2020 policy brief by OECD highlighted the need to create detailed guidelines on procurement strategies during crises including policies for audit and oversight [30]. The World Justice Project published a brief which reinforced the notion that regardless of how expedited procurement processes are, procurement should still be auditable and outline considerations justifying the expedited process [36].

### Barriers and facilitators to the implementation of ACTA mechanisms

Overall, only 5 papers in the academic literature discussed facilitators of and/or barriers to implementation of ACTA mechanisms. In relation to the implementation of standardized monitoring systems, Gnegal (2020) and Moshoeshoe (2022) identified the lack of funding as an important barrier to the implementation of these technologies in low- and middle-income countries [25, 27]. Moeshoeshoe (2022) highlighted the importance of involvement of international stakeholders such as the World Health Organization and UNODC to make implementation possible in resource-limited nations [27]. An additional factor that Moshoeshoe (2022) identified as a barrier to implementing screening technologies is the immature regulatory framework that exists in low and middle-income countries such as those in Sub-Saharan Africa [27].

Several papers identified increased transparency in procurement of health products as a mechanism to reduce corruption within health systems. Within this literature, Abbasi (2020) acknowledged a barrier for implementation of transparency mechanisms is the need to accelerate availability of diagnostics and treatments to support innovation [32]. None of the mentioned articles studied the implementation of proposed ACTA mechanisms and therefore did not discuss barriers to implementation.

## Discussion

In this rapid review, we aimed to identify factors and mechanisms related to corruption surrounding COVID-19 vaccines, medicines, and PPEs. The following are key findings from the rapid review: (1) conditions created by the COVID-19 pandemic have increased opportunities for corruption, specifically in the form of substandard and falsified medicines; (2) strong healthcare systems and structures are instrumental for preventing and addressing corruption making some low- and middle- income countries are more vulnerable to corruption during health emergencies 3) though the potential of ACTA mechanisms has been discussed for preventing and addressing corruption, more research is needed surrounding effective implementation of ACTA mechanisms to minimize corruption during health crises. These collective findings support a call to action for policymakers and other stakeholders to take actionable measures in strengthening regulatory oversight related to pharmaceuticals and health products.

First, findings from the review highlight that the combination of the increased demand and global shortages of vaccines, medicines, and PPE led to exacerbation of corruption risks allowing for the emergence of substandard and falsified medicines during the COVID-19 pandemic. The majority of papers included in this review identified incidents of substandard and falsified products. Four of the papers attributed the emergence of falsified chloroquine in Africa to the increased demand for these medications globally due to off-label use for the treatment of COVID-19 which subsequently led to shortages and increased market prices [25, 38]. This finding aligns with the WHO’s documentation of constrained access to quality and safe medical products during the COVID-19 pandemic [41].

The increase in demand for chloroquine/hydroxychloroquine resulted from misinformation claiming that the off-label use of these medications may be useful in the prevention and treatment of COVID-19 thus leading to increased demand and hoarding [39]. The number of instances of evident falsification of antimalarials in African countries is particularly concerning in the context of malaria treatment in regions with high malaria burden [42].

Substandard medicines contribute to drug-resistant infections as they do not contain the correct active ingredients or do not contain therapeutic amounts thus having detrimental impacts on malaria treatment [41]. A position paper from the United States Pharmacopeia (USP) estimated that pre-pandemic, in just one year, 122,000 children under the age of 5 from 39 sub-Saharan.

African countries lost their lives because of consuming substandard antimalarials [43]. The presence of substandard antimalarials was already widespread pre-pandemic and these impacts are only expected to increase due to the pandemic. While the impacts of the substandard medicines that have emerged during the pandemic on treatment outcomes have not yet been quantified, the number of reports of falsified products seems to indicate an exacerbation of an already prevalent global problem.

A second key finding in our review was the extent of vulnerability amongst low- and middle-income countries to corruption risks during the COVID-19 pandemic. More than half of the papers analyzed in this review referred to corruption in low- and middle-income countries while only 4 discussed incidences of corruption in high-income countries. Sung (2021) and Bracci et al. (2021) identified cases of falsified COVID-19 products being distributed to populations in low- and middle-income countries [44, 45]. Both papers found the emergence of these products to be attributable to under-developed health systems and structures that contributed to limited access to health resources, weak pharmaceutical governance, and limited monetary resources, which are characteristic attributes of many low- and middle-income countries [44, 45]. In addition, Sanchez-Duque et al. (2021) identified vaccine inequity as a factor for emergence of falsified vaccines which is very prevalent in low- and middle-income countries [46]. This finding is not surprising as even in non-crisis times, substandard and falsified medicines are prevalent in low- and middle-income countries [41].

The presence of these substandard health products greatly undermines the collective global COVID-19 response [43]. Lower income countries have unique conditions that put them at risk including weak regulatory oversight and lack of appropriate resources for quality assurance of medicines [43]. While our review did identify more instances of procurement of falsified and substandard products in lower-income countries and less in high-income countries, it is important to note that there are likely more cases in higher income countries that have not been identified.

A targeted search of relevant grey literature reinforced the finding that low- and middle- income countries have been particularly vulnerable to the emergence of substandard and falsified medical products during the COVID-19 pandemic. The grey literature, however, also revealed that no country is immune to the emergence of corruption in times of crises, with cases of substandard and falsified vaccines, PPE, and COVID-19 medicines presented in high-income countries including France, Spain, Italy, Romania, and Slovenia. Cases of substandard and falsified products in these countries involved antiviral medications such as chloroquine used to prevent and treat COVID-19, as well as surgical masks. In comparison, in low- and middle- income countries, cases of falsified products found in this reveal were majority related to COVID-19 vaccines. This finding reveals that low- and middle-income countries were indeed vulnerable to receiving falsified vaccine products because of inequitable vaccine distribution globally.

The third key finding of this rapid review is that there is a need for more research to be done surrounding the effective implementation of anti-corruption, transparency, and accountability mechanisms to prevent corruption during health crises. Of the 20 included studies, 10 discussed potential ACTA mechanisms that could be implemented to reduce corruption risks in the procurement and distribution of COVID-19 vaccines, medicines, and PPE. Potential mechanisms that were explored in these papers included the establishment of appropriate screening technologies that can detect substandard medicines, investment in local production, punitive sanctions for those that sell or distribute falsified products, and transparency in procurement processes for these health supplies. However, the mechanisms discussed were not supported with evidence or examples of real-life implementation. This likely results from the lack of available real-world evidence on effective strategies for combating corruption.

In addition, none of the mentioned studies analyzed the outcomes or benefits of the proposed ACTA mechanisms. More work must be done to study the process of implementation of various anti-corruption mechanisms and measure the impact that it has on parameters that reveal corruption in a region. This research should be carried out in various settings including both low- and high-income countries as the effectiveness of mechanisms may differ.

Transparency International states that the mechanisms of corruption, along with solutions and barriers to reducing these risks, differ greatly between countries, regions, and economic systems and therefore, different strategies are required in each setting [41]. While identifying the presence of and causes of corruption is helpful, it is critical to examine the solutions for improvements and how to tackle arising barriers. While it may be too late to prevent corruption in the landscape of the COVID-19 pandemic, it is important to have these policies and mechanisms in place to reduce corruption in health systems in the event of future health crises.

Guidance notes and policy briefs published by the OECD, World Justice Project, the UNDP, the UNODC, the World Bank Group and Transparency International (April 2020 to April 2021), did not provide clear and specific policies or guidance documents in place for measures for countries to take to minimize corruption risks in procurement and distribution of medicines and supplies during the COVID-19 pandemic. Rather, broad suggestions were provided for preventing corruption in processes during the pandemic that did not account for country-specific contexts and circumstances. Examples of guidance for anti-corruption mechanisms included outlining responsibilities for the oversight of procurement and explicit processes for documentation of transactions, use of e-procurement platforms for transparency and strengthening of audit capacity within institutions. Policies need to be put in place prior to these extreme circumstances so that countries and governments are well prepared and have the appropriate resources available when times of crises strike. Policy briefings published by World Justice Project, UNDP, U4/Transparency International all highlighted that corruption was able to flourish during the COVID-19 pandemic due to a lack of safeguards in transparency and accountability mechanisms and lack of a clear legal framework needed to counter corruption in emergency procurement processes.

### Limitations and strengths

Due to the nature of the accelerated process of a rapid review there are several limitations, mainly that it is possible that some articles were missed. One reviewer was used for screening and data extraction; however, this reviewer was greatly familiar with the topic, and processes, issues, and themes were discussed with the experienced research team. Majority of the papers used in this review identified cases of substandard and falsified products. However, while other forms of corruption such as collusion, embezzlement, and concealment were less prevalent in the literature, the reason for this may be that these acts are more difficult to identify rather than falsified products which can be identified using analytical methods. Despite the limitations of this study design, this rapid review provides relevant evidence that highlights the importance of improving anti-corruption mechanisms. This literature review addresses a crucial gap in the literature on corruption in the procurement of COVID-19 vaccines, medicines, and PPE and identifies gaps in evidence to guide future research in this area.

## Conclusion

This rapid review examined key literature on corruption related to COVID-19 medicines, vaccines, and PPE. After analyzing 20 relevant academic articles and 17 grey literature pieces, three key findings were identified. First, the high demand, in combination with global shortages of essential medicines and health supplies during the COVID-19 pandemic has exacerbated corruption risks globally. Second, low- and middle-income countries have been particularly vulnerable to corruption during the COVID-19 pandemic secondary to limited access to health resources, limited technical and financial resources for adequate quality assurance, and weak pharmaceutical governance. Lastly, more research is needed surrounding effective anti-corruption, transparency, and accountability mechanisms to minimize corruption during future health crises. These collective findings support a call to action for global health actors to enact policies targeted at reducing corruption while increasing accountability and transparency, specifically in times of health crises. In addition, this rapid review has highlighted substantial gaps in the available evidence and can serve as a guide to future research.

## Data Availability

All search strategies are included in Table 1 and included studies are cited herein. Citations for excluded studies are available on reasonable request from the corresponding author.

## Abbreviations

PPE: Personal protective equipment
WHO: World Health Organization
ACTA: Anti-corruption, Accountability, and Transparency

## References

[1] World Health Organization. Who director-general's opening remarks at the media briefing on COVID-19–11 March 2020. 2020. https://www.who.int. Accessed 31 Mar 2022.

[2] Ayati N, Saiyarsarai P, Nikfar S (2020). Short and long term impacts of COVID-19 on the pharmaceutical sector. DARU J Pharm Sci.

[3] Iyengar KP, Vaishya R, Bahl S, Vaish A (2020). Impact of the coronavirus pandemic on the supply chain in healthcare. Br J Healthc Manage.

[4] World Health Organization. Shortage of personal protective equipment endangering health workers worldwide. 2020. https://www.who.int. Accessed 4 Aug 2022.

[5] Transparency International. Why fighting corruption matters in times of COVID-19. 2020. https://www.transparency.org. Accessed 18 Jan 2022.

[6] Transparency International. What is corruption?(n.d.) https://www.transparency.org.Accessed 18 Jan 2022.

[7] Teremetskyi V, Duliba Y, Kroitor V, Korchak N, Makarenko O (2020). Corruption and strengthening anti-corruption efforts in healthcare during the pandemic of covid-19. Med Leg J.

[8] Forman L, Kohler JC (2020). Global health and human rights in the time of COVID-19: response, restrictions, and legitimacy. J Hum Rights.

[9] Oliveria Silva Luz A, Emergencies: Increasing the opportunities to corruption? Geneva Global Policy Brief, University of Geneva. No 1. 2021. p. 1–8. https://ceje.ch/files/4116/1521/7604/University_of_Geneva_-_GGPB_N1-2021_-_A._Oliveira_Silva_Luz.pdf.

[10] National Academies of Sciences, Engineering, and Medicine; Health and Medicine Division; Board on Health Care Services; Board on Global Health; Committee on Improving the Quality of Health Care Globally. (2018). Crossing the Global Quality Chasm: Improving Health Care Worldwide.

[11] Chang Z, Kohler J. The global fund: anti-corruption, transparency and accountability. 2020. 10.21203/rs.3.rs-135134/v1. 10.1186/s12992-021-00753-w PMC8449911 34537059

[12] Kohler JC, Bowra A (2020). Exploring anti-corruption, transparency, and accountability in the World Health Organization, the United Nations Development Programme, the World Bank Group, and the global fund to Fight AIDS, tuberculosis and malaria. Global Health.

[13] Katharina.kiener-Manu. Anti-corruption module 6 key issues: Transparency as a precondition. 2020. https://www.unodc.org. Accessed 31 Mar 2022.

[14] Vian T (2020). Anti-corruption, transparency and accountability in health: concepts, frameworks, and approaches. Glob Health Action.

[15] Vian T (2008). Review of corruption in the health sector: theory, methods and interventions. Health Policy Plann.

[16] Tricco AC, Langlois EV, Straus SE (2017). Rapid reviews to strengthen health policy and systems: a practical guide.

[17] Haby MM, Chapman E, Clark R, Barreto J, Reveiz L, Lavis JN (2016). What are the best methodologies for rapid reviews of the research evidence for evidence-informed decision making in health policy and practice: a rapid review. Health Res Policy Syst.

[18] Moons P, Goossens E, Thompson DR (2021). Rapid reviews: the pros and cons of an accelerated review process. Eur J Cardiovasc Nurs.

[19] World Health Organization. Medical product alert N°4/2020: Falsified chloroquine (update). 2020. https://www.who.int. Accessed 3 Jul 2022.

[20] World Health Organization. Medical product alert N°2/2022: Falsified DESREM (Remdesivir). 2022. https://www.who.int. Accessed 3 Jul 2022.

[21] World Health Organization. Medical product alert N°4/2021: Falsified remdesivir. 2021. https://www.who.int. Accessed 3 Jul 2022.

[22] World Health Organization. Medical product alert N°2/2021: falsified COVID-19 vaccine BNT162b2. 2021. https://www.who.int. Accessed 3 Jul 2022.

[23] World Health Organization. Medical product alert N°6/2021: Falsified pfizer-biontech COVID-19 vaccine. 2021. https://www.who.int. Accessed 3 Jul 2022.

[24] World Health Organization. Medical product alert N°5/2021: Falsified COVISHIELD vaccine (update). 2021. https://www.who.int. Accessed 3 Jul 2022.

[25] ​Gnegel G, Hauk C, Neci R, Mutombo G, Nyaah F, Wistuba D, Häfele-Abah C, Heide L (2020). Identification of falsified chloroquine tablets in Africa at the time of the COVID-19 pandemic. Am J Trop Med Hyg.

[26] Tesfaye W, Abrha S, Sinnollareddy M, Arnold B, Brown A, Matthew C, Oguoma VM, Peterson GM, Thomas J (2020). How do we combat bogus medicines in the age of the covid-19 pandemic?. Am J Trop Med Hyg.

[27] Moshoeshoe RJ, Enslin GM, Katerere DR (2022). An exploratory assessment of the legislative framework for combating counterfeit medicines in South Africa. J Pharm Policy Pract.

[28] Kohler JC, Wright T (2020). The urgent need for transparent and accountable procurement of medicine and medical supplies in times of COVID-19 pandemic. Jo Pharm Policy Pract.

[29] United Nations Office on Drugs and Crime. Research brief: the impact of COVID-19 on organized crime. http://www.unodc.org. 2021. Accessed 3 Jul 2022.

[30] OECD. Policy measures to avoid corruption and bribery in the COVID-19 response and recovery. www.oecd.org (2020). Accessed on 3 Jul 2022.

[31] Coordinator KRR, et al. Mitigating corruption risks in COVID-19 vaccine rollout. U4 Anti-Corruption Resource Centre. https://www.u4.no (2021). Accessed 3 Jul 2022.

[32] Abbasi K (2020). Covid-19: politicisation, “corruption”, and suppression of science. BMJ.

[33] Mottay L, Le Roux J, Perumal R, Esmail A, Timm L, Sivarasu S, Dheda K (2020). KN95 filtering facepiece respirators distributed in South Africa fail safety testing protocols. S Afr Med J.

[34] Jarrett S, Wilmansyah T, Bramanti Y, Alitamsar H, Alamsyah D, Krishnamurthy KR, Yang L, Pagliusi S (2020). The role of manufacturers in the implementation of global traceability standards in the supply chain to combat vaccine counterfeiting and enhance safety monitoring. Vaccine.

[35] Srivastava K (2021). Fake covid vaccines boost the black market for counterfeit medicines. BMJ.

[36] World Justice Project. Corruption and the COVID-19 pandemic. 2020. https://worldjusticeproject.org. Accessed 3 Jul 2022.

[37] UNDP. Transparency, accountability and Anti-corruption service offer for COVID-19 response and recovery. 2020. https://www.undp.org. Accessed 3 Jul 2022.

[38] Waffo Tchounga CA, Sacre PY, Ciza P, Ngono R, Ziemons E, Hubert P, Marini RD (2021). Composition analysis of falsified chloroquine phosphate samples seized during the COVID-19 pandemic. J Pharm Biomed Anal.

[39] Belayneh A (2020). Off-label use of chloroquine and hydroxychloroquine for covid-19 treatment in Africa against who recommendation. Res Rep Trop Med.

[40] World Bank. Ensuring integrity in the government response to covid-19. 2020. https://openknowledge.worldbank.org. Accessed 3 Jul 2022.

[41] World Health Organization. Substandard and falsified medical products. 2018. https://www.who.int. Accessed 31 Mar 2022.

[42] World malaria report. https://reliefweb.int (2021). Accessed on 31 March 2022

[43] USP. SP global public policy position: combatting substandard and falsified medicines. 2020. https://www.usp.org . Accessed 31 Mar 2022.

[44] Sung M, Huang Y, Duan Y, Liu F, Jin Y, Zheng Z (2021). Pharmaceutical Industry’s engagement in the global equitable distribution of covid-19 vaccines: Corporate social responsibility of EUL Vaccine developers. Vaccines.

[45] Bracci A, Nadini M, Aliapoulios M, McCoy D, Gray I, Teytelboym A, Gallo A, Baronchelli A (2021). Dark web marketplaces and covid-19: Before the Vaccine. EPJ Data Sci.

[46] Sánchez-Duque JA, Su Z, Rosselli D, Chica-Ocampo MC, Lotero-Puentes MI, Bolaños-Portilla AM, Dhawan M, Rodríguez-Morales AJ, Dhama K (2021). The ignored pandemic of public health corruption: a call for action amid and beyond SARS-COV-2/COVID-19. J Exp Biol Agric Sci.

